# Difusão espaço-temporal do sarampo: uma análise intraurbana no Rio de Janeiro, Brasil

**DOI:** 10.1590/0102-311XPT166724

**Published:** 2025-02-07

**Authors:** Larissa Nunes Moreira Reis, Alexandre San Pedro, Jefferson Pereira Caldas dos Santos, Yasmin Toledo Santos, Heitor Levy Ferreira Praça, Paula Barbosa Conceição, Gerusa Gibson

**Affiliations:** 1 Instituto de Estudos em Saúde Coletiva, Universidade Federal do Rio de Janeiro, Rio de Janeiro, Brasil.; 2 Instituto de Tecnologia em Fármacos, Fundação Oswaldo Cruz, Rio de Janeiro, Brasil.; 3 Programa de Mestrado Profissional em Meio Ambiente, Universidade Veiga de Almeida, Rio de Janeiro, Brasil.

**Keywords:** Sarampo, Análise Espacial, Vigilância Epidemiológica, Measles, Spatial Analysis, Epidemiologic Surveillance, Sarampión, Análisis Espacial, Vigilancia Epidemiológica

## Abstract

Os objetivos foram analisar o perfil dos casos de sarampo no Município do Rio de Janeiro, Brasil, entre 2007 e 2021 e descrever a difusão e formação de *cluster* nos anos epidêmicos. Realizou-se um estudo ecológico, cujas unidades foram os bairros. Foi conduzida análise bivariada das variáveis socioeconômicas e epidemiológicas, segundo vacinação prévia e ocorrência de hospitalização. As análises de *cluster* e de difusão espacial foram realizadas pelo método de varredura SCAN e de Interpolação pela Ponderação do Inverso da Distância. Dos 774 casos confirmados, 57,6% eram do sexo masculino, 72,9% eram adultos e 63,7% foram identificados como brancos. Observou-se maior proporção de vacinação prévia em brancos, assim como na faixa de 5 e 11 anos. A proporção de vacinação anterior foi maior no estrato de maior escolaridade. Cerca de 16,3% foram hospitalizados, sendo o maior risco entre casos na faixa de 5 e 11 anos e menor entre 18 e 29 anos, quando comparadas às crianças com até um ano de idade. Os primeiros casos da epidemia ocorreram na região da Grande Tijuca, passando a se disseminar em bairros das zonas Oeste e Sul da cidade, revelando difusão hierárquica por realocação. Os conglomerados de maior risco foram formados nas zonas Sul e Centro. Os achados confirmam a mudança no perfil epidemiológico da doença assim como um padrão de difusão influenciado pela hierarquia intraurbana da cidade, onde bairros de maior comércio e turismo atuam como polos iniciadores e difusores da transmissão. Tais aspectos devem fundamentar as estratégias de controle, orientando campanhas de imunização que contemplem as faixas etárias mais acometidas, com ações diferenciadas nas áreas de difusão da doença.

## Abstract (en)

This study aimed to analyze the profile of measles cases in the municipality of Rio de Janeiro, Brazil, from 2007 to 2021 and to describe their diffusion and cluster formation in epidemic years. Neighborhoods were considered as the units of this ecological study. A bivariate analysis of socioeconomic and epidemiological variables was conducted according to previous vaccination and hospitalization. Cluster and spatial diffusion analyses were performed by the SCAN screening method and Inverse Distance Weighting. Of the 774 confirmed cases, 57.6% were men, 72.9% were adults, and 63.7% self-identified as white. A higher proportion of previous vaccination occurred in white individuals and in those who were aged from 5 to 11 years. The stratum with higher schooling showed a higher proportion of previous vaccination. About 16.3% underwent hospitalization, with the highest risk occurring in those aged from 5 to 11 years and the lowest for those aged from 18 to 29 years old when compared to children up to one year of age. The first cases in the epidemic occurred in the Greater Tijuca area, then spreading to western and southern zones, showing hierarchical diffusion by relocation. The highest risk clusters were formed in the south and center zones. Findings confirm the change in the epidemiological profile of the disease and its diffusion pattern under the influence of the intra-urban hierarchy of the municipality, the neighborhoods of which with greater commerce and tourism act as initiators and diffusers of transmission. Such aspects should support control strategies, guiding immunization campaigns that address the most affected age groups and with custom actions in the areas of disease diffusion.

## Resumen

Los objetivos fueron analizar el perfil de casos de sarampión en el municipio de Río de Janeiro, Brasil, entre 2007 y 2021, así como describir la propagación y agrupación en años epidémicos. Se realizó un estudio ecológico cuyas unidades eran los barrios. Se aplicó un análisis bivariado de las variables socioeconómicas y epidemiológicas, según vacunación previa y ocurrencia de hospitalización. Los análisis de clúster y propagación espacial se realizaron utilizando el método de barrido SCAN y la interpolación mediante ponderación de distancia inversa. De los 774 casos confirmados, el 57,6% eran del sexo masculino, el 72,9% en adultos y el 63,7% en personas blancas. Se observó una mayor proporción de vacunación previa en las personas blancas, así como en el grupo etario de entre 5 y 11 años. La proporción de vacunación anterior fue mayor cuanto mayor el nivel de estudios. Aproximadamente un 16,3% de los participantes estuvieron hospitalizados, con un mayor riesgo entre los casos en el grupo etario de entre 5 y 11 años y un menor riesgo en aquellos de entre 18 y 29 años en comparación con los niños de hasta un año de edad. Los primeros casos de la epidemia surgieron en la región del Gran Tijuca, para después extenderse por los barrios de las zonas Oeste y Sur de la ciudad, lo que revela una propagación jerárquica por reubicación. Los conglomerados de mayor riesgo se formaron en las zonas Sur y Centro. Los hallazgos confirman el cambio en el perfil epidemiológico de la enfermedad, así como un patrón de difusión influenciado por la jerarquía intraurbana de la ciudad, donde los barrios con más comercio y turismo fueron los responsables del inicio y la propagación de la transmisión. Estos aspectos permiten ayudar las estrategias de control al orientar las campañas de inmunización que atiendan a los grupos etarios más afectados, con acciones diferenciadas en las áreas de propagación de la enfermedad.

Apesar de ser uma doença prevenível por meio de uma vacina segura, eficaz e amplamente ofertada em todo mundo, o recrudescimento do sarampo tem sido um fenômeno observado em nível global ^1^ ^,^ ^2^.

A transmissão da doença foi considerada eliminada nas Américas em 2016, porém, em 2017, o vírus ressurgiu, ocasionando surtos associados à queda nas coberturas vacinais em diversos países, o que culminou na perda da certificação de região livre da doença ^3^ ^,^ ^4^ ^,^ ^5^ ^,^ ^6^.

No Brasil, foram registradas diversas epidemias, motivando a realização de campanhas de vacinação ^5^ ^,^ ^7^ ^,^ ^8^. O primeiro surto no país nesse período iniciou em 2018, em Roraima, com dispersão para o Amazonas e Pará, resultando em mais de 10 mil notificações e 12 óbitos, 8 deles em menores de 5 anos ^8^. Em 2019, o Estado de São Paulo vivenciou uma epidemia com mais de 20 mil casos confirmados, com dispersão para estados vizinhos ^1^ ^,^ ^3^.

Considerando a relevância da reemergência do sarampo em território nacional e a necessidade de compreensão da dinâmica espacial e temporal de transmissão da doença em escala intramunicipal, este estudo teve como objetivo analisar o perfil dos casos de sarampo e o padrão de difusão espaço-temporal na cidade do Rio de Janeiro.

## Material e métodos

Estudo ecológico, com abordagem espaço-temporal, baseado em casos de sarampo confirmados entre residente do Município do Rio de Janeiro no período de 2007 a 2021. O Município do Rio de Janeiro localiza-se na Região Sudeste do Brasil, tem extensão territorial de 1.182km^2^ e população estimada, em 2022, de 6.211.223 habitantes ^9^, sendo divido em 10 áreas de planejamento, 34 regiões administrativas e 160 bairros, os quais serão as unidades de análise deste estudo ^10^. Foram testadas as diferenças de proporção de vacinação prévia dos casos, segundo variáveis epidemiológicas e socioeconômicas, considerando os valores de p < 0,05 estatisticamente significativos. Também foram obtidas razões de risco (RR) de hospitalização, estratificadas pelas variáveis sexo, faixa etária, raça/cor e situação vacinal prévia, com os respectivos intervalos de 95% de confiança (IC95%). As análises foram realizadas no software R, usando o pacote *EpiR* (https://www.r-project.org/). A identificação de áreas de *clusters* de casos foi restrita ao biênio epidêmico 2019-2020, na qual se utilizou o método de varredura SCAN ^11^. As análises foram realizadas no software SaTScan (http://www.satscan.org), assumindo como parâmetros o modelo discreto de Poisson e tamanho máximo de janela geográfica igual a 5% da população sob risco. Para os conglomerados estatisticamente significativos, foi construído um mapa temático com os riscos relativos. Na análise da difusão espacial do biênio epidêmico (2019-2020), empregou-se a base cartográfica com localização geográfica nas sedes dos bairros, considerando que a localização dos centroides pelo método geométrico pode acarretar perda de precisão, com a localização do centroide em regiões desabitadas. O método de Interpolação pela Ponderação do Inverso da Distância (*Inverse Distance Weighting* - IDW) ^12^ foi utilizado para análise da difusão espacial dos primeiros casos confirmados em cada bairro. A interpolação foi realizada usando a malha pontual dos centroides dos bairros, acrescida das informações das datas de início de sintomas do primeiro caso confirmado e do número de dias com notificação durante o período. Os bairros que não apresentaram casos confirmados não participaram da interpolação e foram identificados no mapa com a cor cinza. Adicionalmente, foi utilizado o número de dias que o bairro apresentou novos casos como parâmetro na interpolação, visando enfatizar o papel daqueles que se mantiveram na função difusora ao longo do tempo. As análises foram realizadas no software QGIS 3.18.1 (https://qgis.org/en/site/). Este estudo foi aprovado pelos comitês de ética em pesquisa do Instituto de Estudos em Saúde Coletiva da Universidade Federal do Rio de Janeiro (parecer nº 6.609.984) e da Secretaria Municipal do Rio de Janeiro (parecer nº 6.628.853).

## Resultados

Entre 2007 e 2021, foram confirmados 774 casos de sarampo, com pico de transmissão em 2019 e 2020, que concentraram 96,6% dos casos do período, gerando 116 (16,3%) hospitalizações. A maioria dos casos era do sexo masculino (57,6%), de adultos jovens na faixa de 18 a 49 anos (72,9%), identificados como brancos (63,7%). Observou-se proporção de vacinação prévia maior entre brancos, quando comparados a pretos, pardos, amarelos e indígenas, assim como na faixa de 5 a 11 anos, quando comparado aos demais grupos etários (p < 0,001). Grande parte dos casos era autóctone (91,4%), e cerca de 42,5% tinham registro de vacinação prévia. Entre os maiores de 18 anos, a proporção de vacinação prévia foi maior no estrato de maior escolaridade (13 anos ou mais), quando comparado às demais categorias (p < 0,01). Na categoria de menor escolaridade (9 anos ou menos), apenas 7,7% tinham registro de vacinação anterior (Tabela 1).

**Tabela 1:** Características epidemiológicas e socioeconômicas dos casos de sarampo confirmados em residentes do Município do Rio de Janeiro, Brasil, no período de 2007 e 2021.

Variáveis	n (%)	Histórico de vacinação		Valor de p
		Sim	Não	
		n (%)	n (%)	
Sexo (n = 774)				0,9506
Masculino	446 (57,6)	190 (42,6)	130 (29,1)	
Feminino	328 (42,4)	139 (42,4)	118 (36,0)	
Faixa etária (anos) (n = 774)				< 0,001
50 ou mais	29 (3,6)	7 (24,1)	10 (34,5)	
30-49	129 (16,7)	50 (38,8)	36 (27,9)	
18-29	435 (56,2)	205 (47,1)	113 26,0)	
12-17	35 (4,5)	16 (45,7)	11 (31,4)	
5-11	18 (2,3)	10 (55,6)	2 (11,1)	
1-4	51 (6,6)	25 (49,0)	19 (37,3)	
< 1	77 (9,9)	16 (20,8)	57 (74,0)	
Escolaridade (anos) (n = 276) *				< 0,001
< 4	26 (9,4)	2 (7,7)	13 (50,0)	
5-9	16 (5,8)	4 (25,0)	9 (56,3)	
10-12	108 (39,1)	61 (56,5)	25 (23,1)	
13 e mais	126 (45,7)	72 (57,1)	27 (21,4)	
Raça/Cor registrada (n = 581)				< 0,001
Branco	370 (63,7)	184 (49,7)	102(27,6)	
Pardo	165 (28,4)	61 (37,0)	65 (39,4)	
Preto	37 (6,4)	17 (45,9)	12 (32,4)	
Amarelo	7 (1,2)	2 (28,6)	4 (57,1)	
Indígena	2 (0,3)	0	1 (50,0)	
Origem da transmissão (n = 613)				< 0,01
Importado	53 (8,6)	38 (71,7)	8 (15,1)	
Autóctone	560 (91,4)	217 (38,8)	203 36,3)	
Critério diagnóstico (n = 772)				0,5478
Laboratorial	573 (74,2)	237 (41,4)	193 (33,7)	
Epidemiológico	185 (24,0)	85 (45,9)	55 (29,7)	
Clínico	14 (1,8)	6 (42,9)	0	
Hospitalização (n = 713)				< 0,001
Não	597 (83,7)	276 (46,2)	185 (31,0)	
Sim	116 (16,3)	30 (25,9)	48 (41,4)	

* Considerados apenas casos com 18 anos e idade ou mais.

O risco de hospitalização foi cerca de duas vezes maior (RR = 2,03; IC95%: 1,12-3,69) na faixa de 5 a 11 anos de idade, quando comparado aos menores de um ano. Por outro lado, o grupo etário de 18 a 29 anos apresentou risco de hospitalização menor (RR = 0,33; IC95%: 0,20-0,54), quando comparado aos casos com menos de um ano. Quando comparados os grupos com e sem registro de vacinação anterior, observou-se que o risco de hospitalização entre não vacinados foi cerca de duas maior vezes (Tabela 2).

**Tabela 2:** Risco de hospitalização segundo características epidemiológicas e socioeconômicas dos casos de sarampo. Município do Rio de Janeiro, Brasil, 2007 a 2021.

Variáveis	n	Hospitalização		RR	IC95%
		n	Risco hospitalar		
Sexo (n = 713)					
Feminino	414	58	14,0	Referência	
Masculino	299	58	19,4	1,38	0,99-1,93
Faixa etária (anos) (n = 713)					
< 1	69	18	26,1	Referência	
1-4	47	20	42,6	1,63	0,97-2,74
5-11	17	9	52,9	2,03	1,12-3,69
12-17	32	8	25,0	0,96	0,47-1,97
18-29	400	34	8,5	0,33	0,20-0,54
30-49	121	18	14,9	0,57	0,32-1,02
50 ou mais	27	9	33,3	1,28	0,66-2,48
Raça/Cor (n = 536)					
Branco	343	44	12,8	Referência	
Pardo	152	38	25,0	1,95	1,32-2,88
Preto	33	6	18,2	1,42	0,65-3,08
Outros *	8	1	12,5	0,97	0,15-6,22
Histórico de vacinação (n = 539)					
Sim	306	30	9,8	Referência	
Não	233	48	20,6	2,10	1,38-3,21

IC95%: intervalo de 95% de confiança; RR: razão de risco.

Nota: excluídos casos com informação de hospitalização ignorada.

* Outros = indígenas e amarelos.

**Figura 1:**
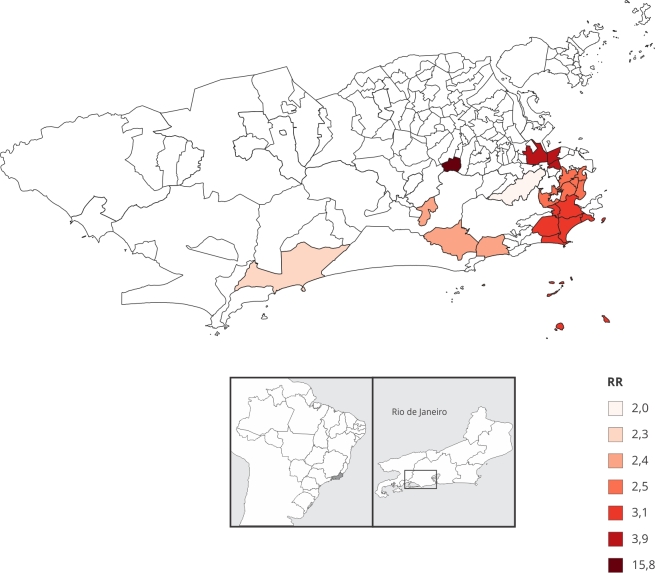
Distribuição espacial dos *clusters* de alto risco de sarampo segundo bairros do Município do Rio de Janeiro, Brasil, no biênio epidêmico de 2019-2020.

Foram identificados oito conglomerados de casos nas Regiões de Planejamento das zonas Sul, Centro, Norte (Tijuca e Méier) e Oeste (Barra da Tijuca) (Figura 1).

RR: razão de risco.

O *cluster* principal foi composto pelo bairro de Água Santa, com uma população estimada de 10 mil habitantes e taxa de incidência de 168,8/100 mil habitantes. Para os demais conglomerados, a população total contida nos *clusters* (937.200 pessoas) representou 15,1% dos habitantes da cidade do Rio de Janeiro, com taxas de incidência variando de 21,3 a 41,7/100 mil habitantes e riscos relativos de 2,0 a 3,9 (Tabela 3).

**Tabela 3:** Características dos conglomerados de alto risco de sarampo segundo bairros do Município do Rio de Janeiro, Brasil, no biênio epidêmico de 2019-2020.

***Clusters*/Bairros**	População	Casos	Incidência *	RR	Valor de p
Água Santa	10.051	17	168,8	15,8	0,000
Santo Cristo/Cidade Nova/São Cristóvão	40.648	17	41,7	3,91	0,001
Botafogo/Humaitá/Copacabana/Leme/Ipanema/Lagoa	322.597	99	30,6	3,09	0,000
Santa Teresa/Flamengo/Glória/Laranjeiras/Catete	168.815	44	26,0	2,48	0,000
São Conrado/Anil/Itanhangá	95.829	24	25,0	2,35	0,050
Recreio dos Bandeirantes	145.383	36	24,7	2,34	0,002
Tijuca	163.928	35	21,3	2,01	0,040

RR: razão de risco.

* Por 100.000 habitantes.

**Figura 2:**
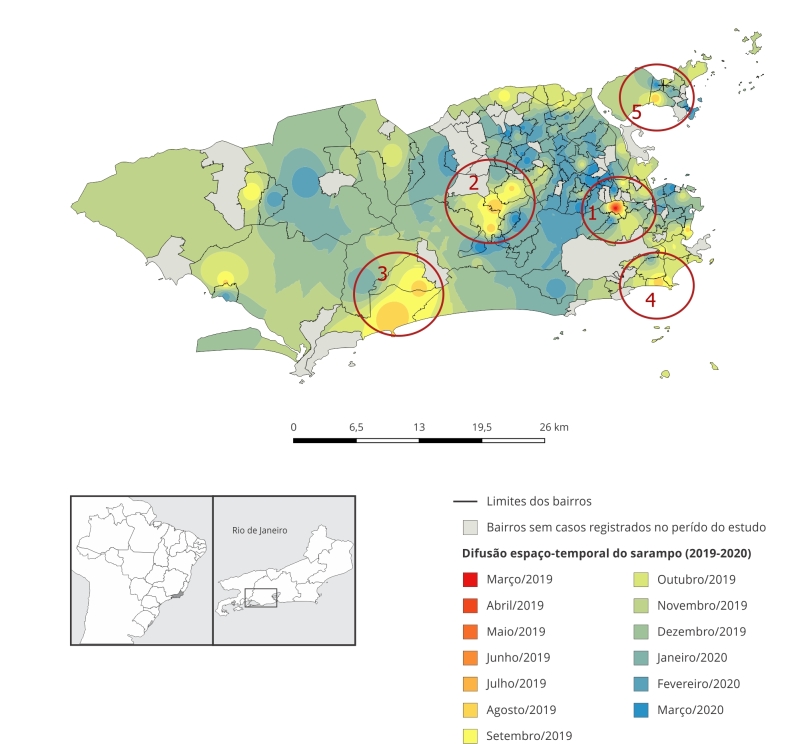
Difusão espaço-temporal de casos de sarampo segundo data de aparecimento dos primeiros sintomas e bairros do Município do Rio de Janeiro, Brasil, entre 2019 e 2020.

Pode-se observar a ocorrência de quatro centros difusores na epidemia. Os primeiros casos de 2019 ocorreram nas Semanas Epidemiológicas 10-16 (março), entre residentes da região da Grande Tijuca (Zona Norte), onde ficou circunscrita até a semana 23 (Figura 2; destaque área 1). Posteriormente, nas Semanas Epidemiológicas 24-35, a epidemia passou a se disseminar nas áreas de Jacarepaguá e bairros associados (Figura 2; destaque área 2), Recreio dos Bandeirantes e Vargem Pequena (Zona Oeste) (Figura 2; destaque área 3), seguido de Ipanema e adjacências (Zona Sul) (Figura 2; destaque área 4).

## Discussão

Os achados deste estudo revelam aspectos importantes do ressurgimento do sarampo no Município do Rio de Janeiro. O perfil observado dos casos mostrou-se semelhante ao dos acometidos na epidemia de São Paulo em 2019, com maior frequência na faixa etária de adultos, em indivíduos com maior escolaridade e da raça/cor branca ^1^. Em 2018, foi instituída a vacinação com dose adicional de tríplice viral em crianças de 6 a 11 meses (Dose Zero) para municípios em risco de surto, estratégia que pode ter servido como proteção em menores de um ano, deslocando o risco de hospitalização para crianças na faixa etária escolar ^13^. Destaca-se ainda o fato de quase metade dos casos apresentarem registro de vacinação anterior contra sarampo, com maior risco de hospitalização entre não vacinados, o que reforça o efeito protetor da vacina contra a evolução grave da doença ^1^. Sobre esse aspecto, persiste o questionamento do porquê haver tantos casos de sarampo com histórico de vacinação anterior. Um ponto a se considerar é a qualidade da informação, uma vez que não é possível confirmar se os esquemas de vacinação eram completos, já que a ficha de notificação não especifica ^1^. Por outro lado, casos de sarampo em populações com esquemas vacinais completos merecem investigação visando avaliar a eventual efetividade da vacina, seja por problemas na rede de frio ou mesmo pela perda de imunidade com os anos, podendo ser necessárias doses de reforço ^14^ ^,^ ^15^. Um aspecto importante é a possível redução dos títulos de anticorpos ao longo do tempo, especialmente em indivíduos vacinados há muitos anos em contextos de baixa incidência da doença (ausência de *booster* natural) ^16^.

Apesar de a maioria dos casos serem identificados como brancos, pretos e pardos tiveram as menores frequências de registro de vacinação prévia, o que sugere desigualdades raciais de acesso a esse recurso ^17^. Como consequência, pardos apresentaram risco significativamente maior de hospitalização, quando comparados aos brancos. Os achados revelaram ainda um padrão de difusão hierárquica por realocação, o que pode ser decorrente de movimentos pendulares de indivíduos entre os bairros, por serem regiões de grande movimentação na cidade conectadas por diferentes transportes modais. Além disso, a região da Tijuca e a Zona Sul são importantes polos comerciais, além de hospedarem um significativo quantitativo de turistas. Tais resultados reforçam a relevância da hierarquia intraurbana no processo de difusão do sarampo e de doenças de transmissão semelhante, podendo condicionar o ritmo de transmissão. Nesse cenário, a incorporação de outras características territoriais, a exemplo da mobilidade urbana e fluxo de turistas, são fundamentais para uma melhor compreensão dos processos de difusão urbana do sarampo ^1^ ^,^ ^18^ ^,^ ^19^. Similarmente, a ocorrência de *clusters* em bairros de parte da Zona Oeste também é influenciada pela nova dinâmica populacional do Município do Rio de Janeiro, marcando a expansão imobiliária e turística, com o desenvolvimento de um importante eixo comercial, especialmente na Barra da Tijuca. Soma-se a isso o elevado poder econômico dos residentes desse bairro e o crescimento da migração no processo de expansão de novas favelas nas adjacências ^20^.

As limitações referem-se, principalmente, à qualidade da informação do sistema de informação e à desatualização das estimativas populacionais. No caso da vacinação prévia, é provável que a informação seja, em grande parte, autorreferida, apesar da orientação de checagem da caderneta de vacinação. Já a variável raça/cor, embora seja um campo da ficha de notificação destinado à autodeclaração, é relativamente comum na rotina dos serviços que ela seja preenchida pelo próprio profissional que fez o atendimento. Adicionalmente, devido ao atraso no censo 2020, utilizou-se estimativas intercensitárias baseadas no crescimento populacional entre 2000 e 2010, o que também pode contribuir para imprecisão nas análises.

Em última análise, os achados sobre a mudança no perfil epidemiológico, assim como o padrão de difusão na cidade, são aspectos que devem fundamentar as estratégias de controle, orientando campanhas de imunização que contemplem as faixas etárias mais acometidas focadas em bairros que funcionaram como polo de iniciação e difusão da doença.

## References

[1] Makarenko C, San Pedro A, Paiva NS, Santos JPC, Medronho RA, Gibson G (2022). Measles resurgence in Brazil analysis of the 2019 epidemic in the state of São Paulo. Rev Saúde Pública.

[2] Venkatesan P (2022). Worrying global decline in measles immunisation. Lancet Microbe.

[3] Makarenko C, San Pedro A, Paiva NS, Souza-Santos R, Medronho RA, Gibson G (2022). Identificação de áreas de risco e fatores associados à epidemia de sarampo de 2019 no Estado de São Paulo, Brasil. Cad Saúde Pública.

[4] Escalante G (2019). El retorno del sarampión en las Américas. Rev Méd Urug.

[5] Pamplona YAP, do Nascimento AMV, de Olinda RA, Barbieri CLA, Braga ALF, Martins LC (2023). Spatial analysis of measles vaccination coverage in the State of São Paulo. BMC Public Health.

[6] Conceição PB, San Pedro A, Praça HL, Santos YT, Reis LNM, Gibson G (2024). Stratification of risk areas for measles transmission a systematic review. Rev Panam Salud Pública.

[7] Chaves ECR, Trindade KN, Andrade BFF, Mendonça MHR (2020). Avaliação da cobertura vacinal do sarampo no período de 2013-2019 e sua relação com a reemergência no Brasil. Revista Eletrônica Acervo Saúde.

[8] Pacheco FC, França GVA, Elídio GA, Leal MB, Oliveira C, Guilhem DB (2020). Measles-containing vaccines in Brazil coverage, homogeneity of coverage and associations with contextual factors at municipal level. Vaccine.

[9] Instituto Brasileiro de Geografia e Estatística Cidades. Rio de Janeiro..

[10] Secretaria Municipal da Casa Civil Regiões Administrativas do Rio de Janeiro..

[11] Kulldorff M (1997). A spatial scan statistic. Commun Stat Theory Methods.

[12] Shepard D (1968). Proceedings of the 1968 ACM National Conference.

[13] Coordenação Geral das Doenças Transmissíveis. Departamento de Vigilância de Doenças Transmissíveis.Secretaria de Vigilância em Saúde.Ministério da Saúde (2018). Nota Informativa nº 119/2018.

[14] He H, Chen EF, Li Q, Wang Z, Yan R, Fu J, Pan J (2013). Waning immunity to measles in young adults and booster effects of revaccination in secondary school students. Vaccine.

[15] Coughlin MM, Beck AS, Bankamp B, Rota PA (2017). Perspective on global measles epidemiology and control and the role of novel vaccination strategies. Viruses.

[16] Davidkin I, Jokinen S, Broman M, Leinikki P, Peltola H (2008). Persistence of measles, mumps, and rubella antibodies in an MMR-vaccinated cohort a 20-year follow-up. J Infect Dis.

[17] Hutchins SS, Jiles R, Bernier R (2004). Elimination of measles and of disparities in measles childhood vaccine coverage among racial and ethnic minority populations in the United States. J Infect Dis.

[18] Santos JPC, Praça HL, Pereira LV, Albuquerque HG, Siqueira ASP (2020). A difusão espacial da COVID-19 no Estado do Rio de Janeiro.. Hygeia.

[19] Leiva GC, Sathler D, Orrico RD (2020). Estrutura urbana e mobilidade populacional implicações para o distanciamento social e disseminação da COVID-19. Rev Bras Estud Popul.

[20] Nicola P (2021). A Zona Oeste do Rio de Janeiro como eixo de expansão urbana para habitação de interesse social considerações a partir do Programa Minha Casa Minha Vida em Senador Camará. Dilemas.

